# Thiazolidinedione Derivative Suppresses LPS-induced COX-2 Expression and NO Production in RAW 264.7 Macrophages

**DOI:** 10.22037/ijpr.2019.1100730

**Published:** 2019

**Authors:** Mehrnaz Rezaei, Hossein Ghafouri, Mahmood Reza Aghamaali, Mostafa Shourian

**Affiliations:** a *Department of Biology, Faculty of Science, University of Guilan, University Campus 2, Rasht, Iran.*; b *Department of Biology, Faculty of Science, University of Guilan, Rasht, Iran.*

**Keywords:** Thiazolidinedione, Cyclo-oxygenase-2, Nitric oxide synthase, Nitric oxide, RAW 264.7 cells

## Abstract

The present study was designed to investigate the inhibitory effect of 2,4 bis-[(4-ethoxyphenyl)azo] 5-(3-hydroxybenzylidene) thiazolidine-2,4-dione (TZD-OCH_2_CH_3_) on the cyclo-oxygenase-2 (COX-2) and inducible nitric oxide synthase (iNOS) in RAW 264.7 cells. The effects of TZD-OCH_2_CH_3 _on COX-2 and iNOS mRNA expression in LPS-activated RAW 264.7 cells were detected by real time PCR. Also, to understand structure and substrate specificity, we have utilized molecular docking simulations (AutoDock Vina) and the active residues in the binding pocket were determined from COX-2 and iNOS. The treatment of RAW 264.7 cells with TZD-OCH_2_CH_3 _significantly inhibited LPS-induced COX-2 mRNA expression, corresponding to 46.1% and 61.06% at 30 and 60 μg/mL, respectively. The present study revealed that the TZD-OCH_2_CH_3_ had a little effect on iNOS mRNA expression. Meanwhile, the TZD-OCH_2_CH_3_ also could inhibit the production of NO compared to single LPS-stimulated cell. According to the results obtained, TZD-OCH_2_CH_3 _dramatically suppressed lipopolysaccharide (LPS) induced nitric oxide (NO) production after 24 h, in a concentration-dependent manner with an IC_50_ of 65 μg/mL. Our data suggest that TZD-OCH_2_CH_3_, as a functionally novel agent, inhibits the inflammatory pathway via suppression of COX-2 mRNA expression and also by the inhibition of the iNOS activity. Therefore, this compound could be suggested as a novel therapeutic strategy for inflammation-associated disorders.

## Introduction

Prostaglandin H2 synthase (PGH2) or COX-2, is chiefly responsible for catalyzing the key step in prostaglandins synthesis as an important biological mediator, that triggers all most inflammation features (1-3). The non-selective inhibitors non-steroidal anti-inflammatory drugs (NSAIDs) are used worldwide for control of inflammation and pain (4, 5). A number of epidemiological studies have concluded that the use of aspirin and other NSAIDs may protect us against the formation of gastrointestinal tumors by inhibitory effects on COXs (6-8). Recent studies suggest that this anticancer effect may be the result of inhibition of COX-2 (7). Several families of compounds having selective COX-2 inhibitory potential have been introduced such as celecoxib, etoricoxib, and rofecoxib (9, 10). However, because of some potential adverse effect such as gastric ulcer, gastrointestinal bleeding, and cardiovascular problems associated with NSAIDs, they have been voluntarily withdrawn from the market (11, 12). It is well-known that a number of heterocyclic compounds exhibit a wide range of pharmacological features (13-15). Substituted thiazolidine-2,4-dionesare is one of the most important heterocyclic compounds with multiple applications (16, 17). There are various pharmaceutical compounds containing the thiazolidine capable of undergoing tautomerism that usually involve migration of mobile proton from one site to another within the molecule (16, 18). Due to this feature, thiazolidine and its derivatives as bioactive heterocycles used as scaffolds for novel drug discovery (19). On the other hand, the thaizolidinediones group, as a known basic pharmacophore for various biological profiles such as antibacterial, anti-HIV, antitumor, and antidiabetic (20-22). «Over the past decades a number of thiazolidine were intensively studied for their anti-hyperglycaemic property (23, 24). Besides, it is reported that 3-thiazolidine-4-one derivatives afforded a new scaffold for anti-inflammatory feature (25, 26). Also, (Z)-5-(4-methoxybenzylidene) thiazolidine-2, 4-dione reported as effective pharmacophore for pro-inflammatory cytokines inhibition (27). In addition, one recent study has shown that a series of thiazolidin-4-one derivatives as novel inhibitors of COX-2 (28). The recent success of thiazolidineas inflammatory mediator’s inhibitor has grown considerable attention towards thiazolidine nucleus in the designing of newer anti-inflammatory drugs. Therefore, we investigated the effects of TZD-OCH_2_CH_3_ on changes of COX-2 and iNOS expression in RAW 264.7 macrophage cells, which can be stimulated with LPS to mimic the condition of infection and inflammation. 

## Experimental

Murine monocytic macrophage cell line RAW264.7 cells were purchased from Iranian Biological Resource Center (IBRC). Lipopolysaccharide (*Escherichia coli* 0127: E8) was purchased from Sigma Chemical Co. Dulbecco’s modified essential medium (DMEM) and fetal bovine serum (FBS) were purchased from Gibco BRL (Grand Island, NY, USA). Total RNA extraction (RNeasy mini kit) and cDNA synthesis kit was obtained from Qiagen. SYBR® Green Real-Time PCR Master was purchased from Thermo Fisher Scientific. MTT and all other chemicals were provided by Merck (Germany). T75, T25 flasks, and 96-well plates were distributed by SPL life science (Korea). 

*Chemical synthesis*

2,4 bis-[(4-ethoxyphenyl)azo]5-(3-hydroxybenzylidene) thiazolidine-2,4-dione (TZD-OCH_2_CH_3_) was synthesized by treating the corresponding aryl diazonium salts with 5-(3-hydroxybenzylidene) thiazolidine-2,4-dione in alkaline media using diazotization-coupling reactions, as previously described (29). The structure of TZD-OCH2CH3 was confirmed by ^1^H NMR and FT-IR spectroscopy. IR spectra were recorded on a Shimadzu 8400 FT-IR spectrophotometer. The ^1^H NMR spectra were obtained on a FT-NMR (400 MHz) Brucker apparatus spectrometer. 

*Cell culture*

The cells were maintained in complete DMEM supplemented with 10% fetal bovine serum (FBS), 100 U/mL of penicillin, and 100 μg/mL of streptomycin and 1.5% sodium bicarbonate at 37 °C and 5% CO_2_. They were then transferred to medium containing 10% DMSO, frozen in liquid nitrogen for long-term storage. The cells were plated at a density of 1 × 10^5^ cells/T-25 flask plate for 48 h.

*Cell proliferation assay*

The murine macrophage cell line RAW 264.7 proliferation was evaluated using the MTT assay as described by Scudiero *et al.* (30). For stimulation, the medium was replaced with 0.1% FBS contained DMEM, and the cells were then stimulated with 1 μg/mL of LPS and were treated with various concentrations of TZD-OCH_2_CH_3 _for 24 and 48 h. Some cells were grown in 1% DMSO as a negative control. At the end of treatment period, 50 μL of MTT solution (0.5 mg/mL) was added to each well and the plate was incubated for 3 h. The supernatant were removed and formazan crystals formed were solubilized in 50 μL of DMSO for 30 min. The absorbance at 570 nm was measured using a Multi-Mode Microplate reader (BioTek Winooski, VT, USA). The results were expressed as percentage of the control (considered as 100%).

*Nitrite measurement*

Accumulated nitrite concentration in the cells culture media was measured following Granger *et al* assay with some modifications (31). RAW 264.7 macrophages were seeded at a density of 5 × 10^3^ cells/well and incubated overnight. Then, cells were treated with TZD-OCH_2_CH_3 _at concentrations ranging from 0-60 μg/mL for 24 h at the presence or absence of 1 μg/mL LPS. 100 μL of cells supernatants were collected and treated with 100 μL of Griess reagent and after 10 min incubation at room temperature the absorbance at 570 nm was measured using Multi-Mode Microplate reader. Sodium nitrite (0–100 µM) was used as standard for the generation a calibration curve.

*RNA extraction and Real time PCR*

The mRNA levels of LPS-induce COX-2 and iNOS were determined in RAW246.7 macrophage cells. Total RNA was extracted from RAW246.7 macrophage cells using Qiagen kit according to the manufacturer’s instruction. RAW246.7 macrophage cells were treated with TZD-OCH_2_CH_3 _at concentrations ranging from 20 to 100 μM for 18 h at the presence or absence of 1 μg/mL LPS. cDNA was amplified using real-time PCR (Applied Biosystems™) with the Fast Start DNA Master SYBR Green I kit. The copy number of each transcript was calculated as the relative copy number normalized by GAPDH copy number. Total RNA was converted to cDNA using a Reverse Transcription System (Qiagen). The target cDNA was amplified using the primers listed in Table 1. Briefly, each amplification reaction contained 25 ng of the cDNA, 0.5 μL (10 pmol/μL) of each primer and 6 μL CYBR green real time-PCR master mix. PCR was performed using the following amplification program: Initial denaturation for 3 min at 94 °C, followed by 35 cycles at 94 °C for 10 sec, 56 °C for 30 sec and 72 °C for 30 sec for COX-2 and 35 cycles at 94 °C for10 sec, 56 °C for 30 sec and 72 °C for 30 sec for iNOS. Melting curve was recorded by cooling the PCR product to 60 °C for 30 sec and then slowly heating it to 94 °C at 0.1 °C/sec to ensure absence of nonspecific products.

*Docking*

The feasibility of TZD-OCH_2_CH_3 _to be ligand for COX-2 and iNOS structures was evaluated using molecular docking. Protein structures were obtained from the RCSB Protein Data Bank (3LN1 and 4UX6 respectively). Missing hydrogen atoms were added to the crystal structures by REDUCE program (32) that also reproduces the correct protonation states of histidine residues and optimizes some sort of side chain flexibilities via a sequence of flipping and rotating movements. All docking simulations were performed by AutoDock Vina program (33), while MGLTools (34) was used for the preparation of necessary input files. A standard docking protocol was adopted that includes addition of Gasteiger atomic charges and assignment of default atom-types. In the accurate docking step, the exhaustiveness parameter was set to 1000. 

Ligand structure was model built and optimized by HyperChem program version 7 using amber force filed (35). Images were created using Python Molecule Viewer (PMV) and the program LigPlot‏ v.1.0, which generates schematic 2-D representations of protein-ligand complexes from the PDB file input (36).

*Statistical analysis*

All experiments were done in triplicates and the results were expressed as the mean ± SD. Comparison between groups was made with One-way ANOVA analysis with post hoc Newman–Keuls tests. *P*-values less than 0.05 were considered statistically significant.

## Results

*Effects of TZD-OCH*_2_ *CH*_3_ *on RAW 264.7 cells proliferation*

**Figure 1:**
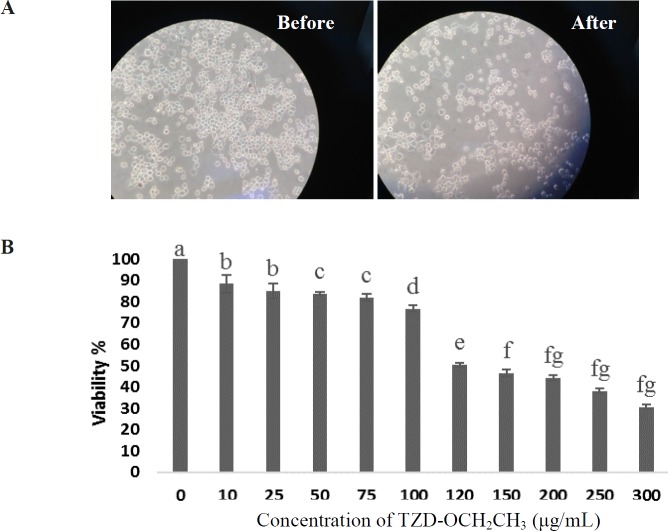
Effect of TZD-OCH2CH3 on cell viability in LPS-stimulated RAW264.7 cells. (A) Before and 24 h after TZD-OCH2CH3 treatment. (B) Cells were incubated in the presence of TZD-OCH2CH3 (0-300 μg/mL) with the addition of 1 µg/mL LPS for 24 h. Cell viability was determined by the MTT assay. Values represent the means ± SDs of three independent experiments. *P < 0.05 indicates statistically significant differences from the control group

We first measured the cytotoxic effect of TZD-OCH_2_CH_3 _on RAW 264.7 cells. The result showed that dimethyl sulfoxide (DMSO) as the solvent of TZD-OCH_2_CH_3 _had no effect on the proliferation of RAW 264.7 cells at concentrations up to 1.5% (not shown results). In following, the viability of the cells stimulated by LPS (1 µg/mL) for 24 h in the presence of TZD-OCH_2_CH_3 _(0-300 µg/mL) was investigated. As shown in Figure 1, the viability of the cells treated for 24 h were not significantly affected by TZD-OCH_2_CH_3 _up to 60 μg/mL compared to the control. Therefore, TZD-OCH_2_CH_3_ concentrations of 30, and 60 μg/mL were used in the subsequent experiments.

*Inhibition of nitrite production by TZD-OCH*_2_ *CH*_3_

To investigate the anti-inflammatory effects of TZD-OCH_2_CH_3_, we tested its effect on NO production in LPS-activated RAW 264.7 cells. 

**Figure 2:**
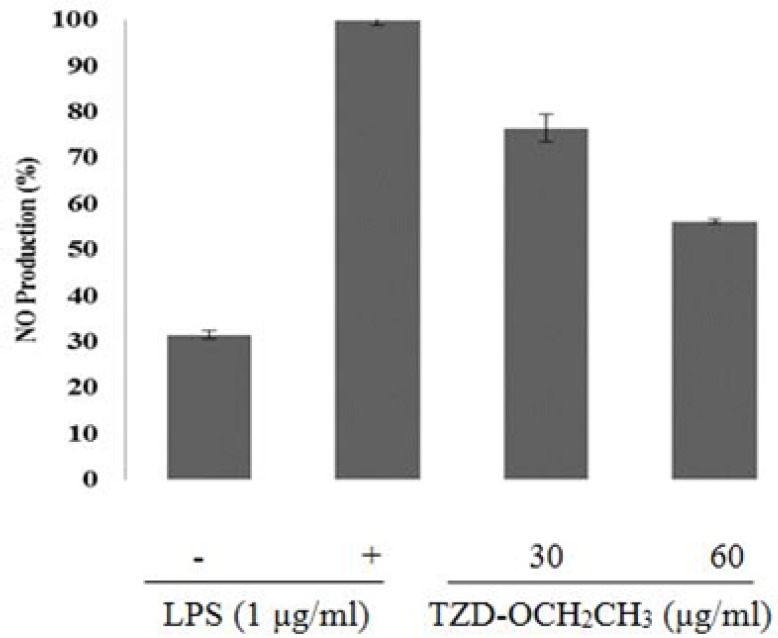
Effect of TZD-OCH2CH3 on LPS-induced NO level in RAW 264.7 cells. The cells were stimulated with 1 μg/mL of LPS only or with different concentrations of TZD-OCH2CH3 for 24 h. NO levels were determined using Griess assays in culture media. Values represent the means ± SDs of three independent experiments. *P < 0.05 indicates statistically significant differences from the control group

As shown in Figure 2, TZD-OCH_2_CH_3 _inhibited nitrite production about 45% at 60 μg/mL. As shown in Figure 2, TZD-OCH_2_CH_3 _was inhibitory effects on the production of NO in LPS-induced RAW 264.7 cells with an IC_50_ of 65 μg/mL in a concentration-dependent manner. LPS treatment of the RAW 264.7 cells increased NO production about 70% over the basal level. 

However, it decreased by 76.4% and 56.2% in the presence of 30 and 60 μg/mL of TZD-OCH_2_CH_3_, respectively, compared with the LPS alone (100%).

*Inhibition of COX-2 and iNOS mRNA expression by TZD-OCH*_2_ *CH*_3_

**Figure 3:**
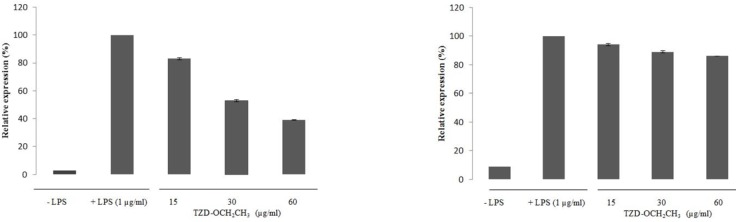
Effect of TZD-OCH2CH3 on the mRNA expression of COX-2 and iNOS in LPS-activated RAW 264.7 cells. RAW 264.7 cells were pretreated with various concentration of TZD-OCH2CH3 (15, 30 and 60 μg/mL) for 1 h before being incubated with LPS (1 μg/mL) for 18 h. Total RNAs were isolated and mRNA expression of COX-2 and iNOS was determined by real-time RT–PCR. Data represent three independent experiments and are expressed as mean ± SDs. *P < 0.05 indicates statistically significant differences from the control group

The effect of TZD-OCH_2_CH_3 _on the expression of COX-2 and iNOS mRNA in LPS-activated RAW 264.7 cells was shown in Figure 3. No any expression of COX-2 and iNOS mRNA was found in unstimulated macrophages while LPS-activated RAW 264.7 cells dramatically induced the COX-2 and iNOS mRNA expression. LPS treatment of the RAW 264.7 cells increased the COX-2 and iNOS mRNA expression about 33.34 and 11.24-fold over the basal level, respectively. The inhibitory effect of TZD-OCH_2_CH_3 _on COX-2 and iNOS mRNA expression at 0-60 μg/mL was in a concentration-dependent manner, indicating that TZD-OCH_2_CH_3 _showed considerable inhibitory effect on the expression of iNOS and COX-2 mRNA in LPS-activated macrophages compared to the control cells. The treatment of LPS-activated RAW 264.7 cells with TZD-OCH_2_CH_3 _significantly inhibited LPS-induced COX-2 mRNA expression, corresponding to 46.1% and 61.06% in the presence of 30 and 60 μg/mL, respectively.

**Table 1:** Sequences of forward and reverse primers

**Primer Sequence**		**Tm (°C)**
COX-2 F ATATCAGGTCATCGGTGGAGAG		64.9
COX-2 R CACTCTGTTGTGCTCCCGAA		65.4
iNOS F GTGCTAATGCGGAAGGTCAT		64.3
iNOS R AAATGTGCTTGTCACCACCAG		64.7

**Figure 4:**
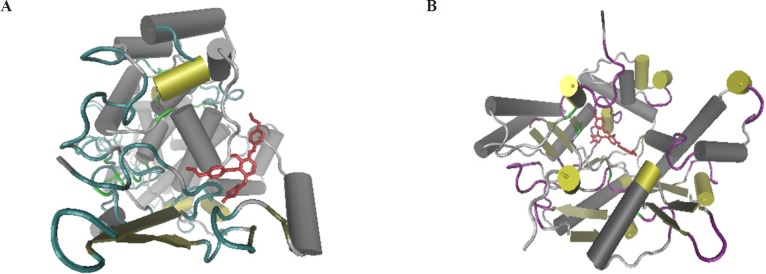
3D secondary structure of COX-2 (A) and iNOS (B) binding site that shows the three dimensional position of TZD-OCH2CH3 at the binding site of the protein

**Figure 5:**
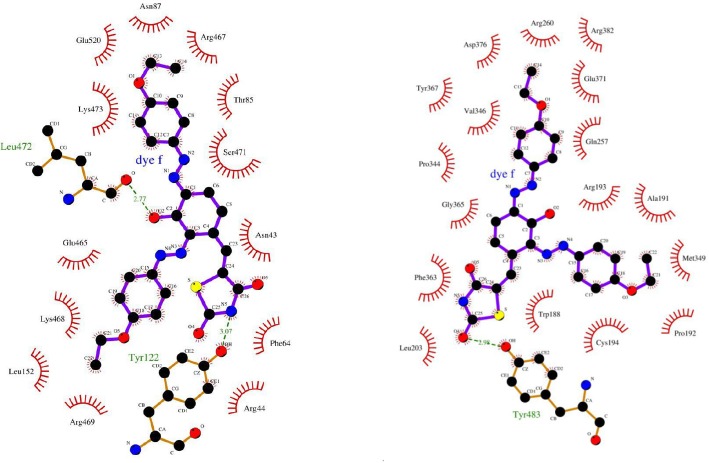
Two dimensional plot of COX-2 (A) and iNOS (B) at the present of TZD-OCH2CH3. LigPlot v.1.0, which generates from the PDB file input

*Docking*

The molecular docking would give the computational insight into the binding of TZD-OCH_2_CH_3_ to COX-2 and iNOS. The results of docking were well clustered around the conformer with the best score. According to Figure 4, the analysis of results showed that hydrophobic interactions play the major role in the binding of TZD-OCH_2_CH_3_ to COX-2 and iNOS hydrophobic pocket. Based on the results of the modeling, dominant interaction is hydrophobic. It is clear from Figure 5A, the active site of COX-2, that interacting with TZD-OCH_2_CH_3_ is composed from Asn43, Arg44, Phe64, Thr85, Asn87, Leu152, Glu465, Arg467, Lys468, Arg469, Ser 471, Lys473, and Glu520. Also, two hydrogen bonds were seen among TZD-OCH_2_CH_3 _and Tyr122 and Leu472 of Cox-2. Figure 5B was shown the Lig Plot diagram of the interaction between iNOS and TZD-OCH_2_CH_3_. Hydrophobic interaction plays the major role in the binding site. 

The residues of binding site are consisted of Trp 188, Ala191, Pro 192, Arg193, Cys194, Leu203, Gln257, Arg260, Pro344, Val346, Met349, Phe 363, Tyr367, Glu 371, Asp376, and Arg382. Besides that, there was on hydrogen bond between Tyr483 of iNOS and TZD-OCH_2_CH_3_. 

## Discussion

Several anti-inflammatory agents such as 2,6-Dimethoxy-4-vinylphenol, cyclicpeptides, and teprenone with anticancer properties have been developed and patented by many pharmaceutical companies (37-39). Despite the fact that there have been many efforts to develop anti-inflammatory agents, there is still a large challenge for developing effective agents (40, 41). 

Therefore, there is an urgent need to develop anti-inflammatory agents with novel mechanisms of action. In an attempt to identify novel anti-inflammatory agents, in present study we investigated the effects of TZD-OCH_2_CH_3 _on LPS-induced expression of iNOS and COX-2. During our search for novel anti-inflammatory agents from synthetic derivatives, we found that thiazolidine exhibited the anti-inflammatory properties; therefore, in this investigation our main objective is design, synthesis, and anti-inflammatory evaluation of a novel derivative of thiazolidineas inhibitors of expression of iNOS and COX-2. Importantly, iNOS and COX-2 has been shown to be responsible for the inflammatory activity and tumorigenesis (42). In RAW 264.7 cells, LPS induces the expression of iNOS, and thus, increases NO production (43). NO is a major macrophage-derived inflammatory mediator and the amount of production of NO may reflect the degree of inflammation (44). 

To investigate the anti-inflammatory activity of TZD-OCH_2_CH_3_ was tested it effect on NO production in LPS-induced RAW 264.7 cells. TZD-OCH_2_CH_3 _inhibited nitrite production by approximately 45% at 60 μg/mL, also the results showed that TZD-OCH_2_CH_3 _was inhibitory effects on the production of NO in a concentration-dependent manner with an IC_50_ of 65 μg/mL. Liang Ma *et al.* (2015) introduced novel derivatives of thiazolidine ((Z)-N-(3-Chlorophenyl)-2-(4-((3-(methoxymethyl)-2,4- dioxothiazolidin-5-ylidene)methyl) phenoxy) acetamide and (Z)-N-(3-Chlorophenyl)-2-(4-((3-(3-dimethylamino)-2-methylpropyl)-2,4-dioxothiazolidin-5-ylidene) methyl) phenoxy) acetamide) that inhibit the production of the NO and the iNOS activity in LPS-induced RAW 264.7 macrophages with an IC_50_ values of 45.6 µM and 25.2 μM, respectively (45). Ma L *et al.* have reported that novel 5-benzylidenethiazolidine-2,4-dione derivatives inhibits iNOS expression in RAW 264.7 cells with an IC_50_ values of 8.66 µM (46). Also, previous studies indicated that the aspirin inhibited the production of the NO with an IC_50_ value of 3.0 mM (47). The COX-2 and iNOS mRNA expression was decreased by treatment of TZD-OCH_2_CH_3 _in the RAW264.7 macrophages compared to the stimulation by LPS alone. The treatment of LPS-activated RAW 264.7 cells with TZD-OCH_2_CH_3_ significantly inhibited LPS-induced COX-2 mRNA expression, corresponding to 37.10% and 67.76% at 30 and 60 μg/mL, respectively. These data suggest that the inhibition of nitric oxide production is not primarily due to a decreased level of iNOS mRNA, but decrease in the production of the NO might be resulted from the inhibition of the iNOS enzyme activity in treated RAW 264.7 cells by TZD-OCH_2_CH_3_. In conclusion, this study showed that TZD-OCH_2_CH_3 _significantly reduced the production of NO and COX-2 and iNOS mRNA expression in LPS-activated RAW 264.7 cells. Our findings suggested that the novel derivative of thiazolidine may be a potent synthetic anti-inflammatory agent.
